# Multifaceted roles of mycobacterial HflX: ribosome splitting, rRNA disordering, and drug resistance

**DOI:** 10.1042/BST20253084

**Published:** 2025-08-20

**Authors:** Soneya Majumdar, Pallavi Ghosh, Rajendra K. Agrawal

**Affiliations:** 1 Division of Translational Medicine, Wadsworth Center, New York State Department of Health, Albany, NY, 12237, U.S.A.; 2 Division of Genetics, Wadsworth Center, New York State Department of Health, Albany, NY 12237, U.S.A.; 3 Department of Biomedical Sciences, University at Albany, Albany, NY, U.S.A.

**Keywords:** ribosome–antibiotic interactions, ribosome structure, rRNA structure and function, structural biology

## Abstract

High frequency of lysogenization X (HflX) is an enigmatic protein that has been implicated in rescuing translationally stalled ribosomes and macrolide-lincosamide antibiotic resistance, as well as in ribosome biogenesis. The protein shows significant sequence and structural variation across species, including variation among paralogs within the same organism. Recent cryo-EM structure determination of ribosome-HflX complexes from different eubacterial species has provided important mechanistic clues to HflX function. Mycobacterial HflXs carry a distinct N-terminal extension (NTE) and a small insertion, as compared with their eubacterial homologs, suggesting that the mycobacterial HflX could have distinct functional mechanisms. This article presents a brief overview of these studies highlighting (i) what we have learned from recent multiple mycobacterial ribosome-HflX complex structures and (ii) the roles of mycobacteria-specific segments in ribosomal RNA disordering that leads to ribosome splitting to rescue translation by removing the drug-bound stalled ribosome from the translationally active polysome pool. Future studies needed to resolve some of the outstanding issues related to HflX function and dynamics are also discussed.

## Introduction

Ribosome recycling, or splitting, is a crucial step in protein synthesis that ensures ribosomal subunits remain available for new translation cycles. In eubacteria and mitochondria this process is facilitated by ribosome recycling factor, and elongation factor G, in its GTP-bound state, mediates the disassembly of post-termination ribosome complexes [1–10]. This recycling is essential for maintaining translational efficiency and ribosome turnover. However, under stress conditions, ribosomes can stall during protein synthesis [11] necessitating specialized rescue mechanisms. One such mechanism involves the universally conserved GTPase, high frequency of lysogenization X (HflX) [12,13].

The* hflX* was first identified in *Escherichia coli* (*Eco*) as part of the *hflA* operon, originally linked to the lytic-lysogeny decision during phage infection [14]. However, subsequent studies revealed that HflX does not significantly affect lambda lysogeny but instead shares homology with the ODN (Obg, DRG1, and Nog1) GTPase family, which plays key roles in ribosome assembly [15–17]. In *Chlamydophila pneumoniae*, HflX was first identified as a GTPase that co-fractionates with ribosomal subunits, providing evidence of its intrinsic GTPase activity and association with the ribosome—observations that have since been confirmed in other bacterial species [18]. That study implied that HflX might play a role in protein synthesis or ribosome assembly.

A previous study in *Eco* [12] demonstrated that *hflX* is regulated by heat shock promoters and that HflX mediates the splitting of translationally stalled, vacant, or mRNA-associated 70S ribosomes with deacylated tRNA at the peptidyl site (P-site) into free 50S and 30S subunits. That study provided the first cryo-EM structure of *Eco* 50S subunits bound to HflX, revealing three distinct domains: an N-terminal domain (NTD), a GTPase domain (GD), and a C-terminal domain (CTD). The NTD comprises two subdomains: ND1, which interacts with helix 69 (H69) of the 23S rRNA, and an α-helical domain (HD), which has a loop that extends into the peptidyl transferase center (PTC). Subsequently, the mammalian mitochondrial homolog of HflX, GTPBP6, was shown to function not only in ribosome splitting *in vitro* and *in vivo* but also in large ribosomal subunit assembly, indicating a dual role [19].

More recently, a second copy of the *hflX* gene, *lmo0762*, was identified in *Listeria monocytogenes* (*Lmo*) and found to be induced in response to lincomycin, unlike the canonical *hflX* gene (*lmo1296*) [20]. Since *lmo0762* was implicated in lincomycin resistance, it was named *hflXr*, distinguishing it from the canonical *hflX* (*lmo1296*) [20]. Unlike *Lmo*, mycobacterial and *Eco* species possess only a single copy of *hflX*. In the context of its genomic location, the mycobacterial *hflX* is more closely related to the canonical *Lmo hflX* (*lmo1296*) than to *Lmo hflXr* (*lmo0762*). Duplications of *hflX* have been observed in species of α-, β-, γ-, and δ-proteobacteria, archaea, and the Firmicutes, thereby highlighting the importance of *hflX*. We speculate that these *hflX* paralogs have evolved to deal with diverse species of stalled ribosomes generated under different stress conditions.

Like *Lmo*, in *Mycobacterium abscessus* (*Mab*) and *Mycobacterium smegmatis* (*Msm*), sublethal concentrations of macrolide-lincosamide antibiotics up-regulate *hflX* expression, yet deletion mutants (ΔMab_*hflX* and ΔMsm_*hflX*) show no change in heat sensitivity, unlike *Eco* [21]. In *Mab*, *hflX*-mediated resistance to macrolide-lincosamide antibiotics is as significant as that conferred by *erm41*, a major macrolide resistance determinant [21]. However, in *Mycobacterium bovis* (*Mbo*), *hflX* was found to play a role in the pathogen’s transition to a non-replicating, drug-tolerant state under hypoxia but was not implicated in antibiotic resistance during aerobic growth [13].

Despite the above-described differences, a common feature among all HflX homologs (HflX and HflXr) is their ability to split stalled, vacant, or deacylated peptidyl-tRNA-bound 70S ribosomes. However, whether HflX’s antibiotic resistance function is dependent on or independent of its ribosome-splitting activity remains unclear. Recent cryo-EM and time-resolved cryo-EM (TRCEM) studies in *Msm* [22], *Eco* [23], and *Lmo* [24] have attempted to elucidate the mechanisms of HflX-mediated 70S splitting and antibiotic resistance. However, the 70S-HflX intermediates captured so far by TRCEM exhibit only subtle conformational changes, offering limited insight into the exact splitting mechanism. Additionally, the absence of structures showing both HflX and drug molecules bound to the ribosome had hindered our understanding of HflX’s role in antibiotic resistance until very recently.

In this review, we summarize the current knowledge of HflX-mediated 70S splitting and antibiotic resistance and highlight how our recent study on *Msm* HflX [25] has reshaped our understanding of both these processes.

## Mechanism of HflX-mediated 70S splitting

Gao and colleagues proposed a plausible mechanism for HflX-mediated 70S ribosome splitting in *Eco* based on a cryo-EM structure of the 50S-HflX complex [12]. Their structure revealed that HflX binding to the 50S ribosomal subunit displaces H69 of the 23S rRNA to accommodate the HflX–ND1 domain. H69 forms a crucial inter-subunit bridge (B2a) between the 50S and 30S ribosomal subunits, henceforth referred to as large ribosomal subunit (LSU) and small ribosomal subunit (SSU), respectively. Its displacement was suggested to destabilize the 70S ribosome, leading to dissociation into LSU and SSU. However, the same study suggested two distinct activities of HflX: an anti-association activity, where HflX binds a pre-dissociated LSU to prevent reassociation with SSU, and a dissociation activity, where HflX actively splits 70S ribosomes into LSUs and SSUs [12]. (The term ‘pre-dissociated LSU’ refers to mature LSU that is about to associate with the SSU to form the 70S ribosome.) Notably, the cryo-EM structure analyzed in that study was derived from a pre-dissociated LSU–HflX complex, raising the possibility that the observed mechanism applies exclusively to the anti-association function rather than ribosome dissociation.

To address this, recent TRCEM studies in *Eco* [23] and *Lmo* [24] analyzed 70S-HflX complexes at different time points. In *Eco*, 70S-HflX–GTP intermediate structures at 10, 25, and 140 milliseconds suggested that HflX binding, combined with the SSU rotation with respect to LSU [26,27], leads to 70S ribosome splitting [23]. In contrast, the *Lmo* study prepared 70S-HflXr complexes in the presence of GDPCP, harvested the complexes at 160 and 600 seconds, and from their structures concluded that HflXr binding does not disrupt bridge B2a directly [24], as previously suggested [12]. Instead, HflXr induces a displacement of the intact bridge B2a toward the SSU, triggering movements in the SSU platform that ultimately disrupt inter-subunit bridges at the platform [24]. These studies collectively suggest that HflX-mediated 70S splitting involves a series of subtle inter-ribosomal subunit conformational changes.

**Figure 1:**
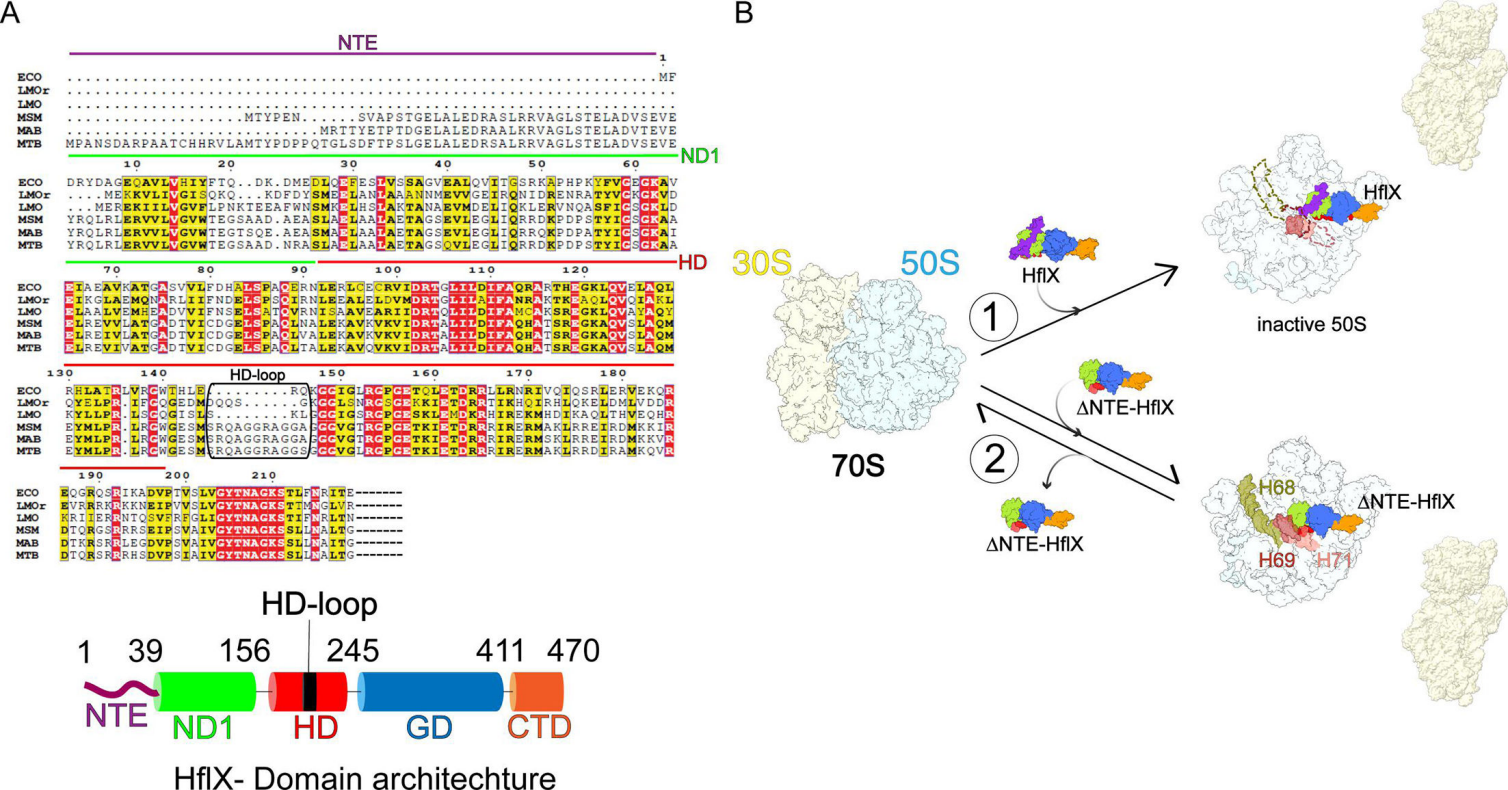
The mycobacteria-specific N-terminal extension in HflX confers a distinct mechanism for splitting stalled 70S ribosomes. (**A**) Multiple sequence alignment of the N-terminal domains (ND) from well-studied select bacterial HflX homologs: *Escherichia coli* (ECO), *Listeria monocytogenes lmo0762* (LMOr), *Listeria monocytogenes lmo1296* (LMO), *Mycobacterium smegmatis* (MSM), *Mycobacterium abscessus* (MAB), and *Mycobacterium tuberculosis* (MTB). The mycobacteria-specific N-terminal extension (NTE, purple) and the HD-loop insertion (black box) are highlighted. A schematic of the overall domain architecture of mycobacterial HflX is shown (bottom), with individual domains color-coded as follows: NTE (purple), N-terminal domain 1 (ND1, green), helical domain (HD, red), HD-loop (black), GTPase domain (GD, cornflower blue), and C-terminal domain (CTD, orange). (**B**) Mechanistic model for *Msm* HflX-mediated 70S ribosome splitting into two ribosomal subunits. Pathway 1: *Msm* HflX-NTE induces disordering (depicted as dotted lines) of multiple 23S rRNA helices—H68 (olive green), H69 (brown), and H71 (salmon)—during 70S splitting. Pathway 2: in the absence of the NTE, 70S splitting is less efficient and occurs without disordering of these rRNA helices, indicating a direct role for the NTE in promoting the disordered state. H68, helix 68; H69, helix 69; H71, helix 71; HflX, high frequency of lysogenization X.

However, whether such a ribosome-splitting mechanism is conserved across bacteria remains unclear, particularly in mycobacteria, where ribosomes contain two additional inter-subunit bridges formed by small ribosomal proteins bS6 and bS22 interacting with helices H54a and H70 [28]. Furthermore, HflX exhibits species-specific variations (Figure 1A). Mycobacterial HflX contains N-terminal extensions (NTEs) of varying lengths—39 amino acids in *Msm* and *Mab*, and 58 amino acids in *Mycobacterium tuberculosis* (*Mtb*)—as well as a nine-amino acid insertion in the HD-loop. HflX with NTE, while predominantly observed in actinobacteria, is also present in several *Clostridium* spp. and *Paenibacilli* spp. However, the presence of extended HD-loops is largely restricted to Actinobacteria, including mycobacteria.

Recently, we demonstrated that *Msm* HflX employs a distinct 70S ribosome splitting mechanism [25]. HflX, in its GTP-bound state, is known to bind vacant or deacylated peptidyl-tRNA-bound 70S ribosomes [12]. Our *Msm* 70S-HflX structure provides insight into the initial recognition and accommodation of HflX on the ribosome. The binding process involves initial interactions through the HD, GD, and CTD domains. The HD extends toward the PTC, likely sensing the acylation state of the peptidyl-tRNA, while the ribosomal L7/L12 stalk base (Sb) engages with GD. Additionally, the Switch-I-loop of GD, which remains ordered only in the GTP-bound state, interacts with the 23S rRNA helix 89, possibly confirming HflX’s active conformation. Once these prerequisites are met, SSU head rotation transitions the P-site tRNA into a P/E hybrid state, facilitating HflX–ND1 accommodation and the concomitant interaction of HflX–CTD with the Sb. HflX–ND1 accommodation then displaces H69, disrupting the inter-subunit bridge B2a and initiating the process of 70S splitting.

Unlike the post-dissociated 50S-HflX complexes observed in *Eco* [23] and *Lmo* [24], our study identified (i) a large pool of inactive 50S ribosomal subunits with multiple 23S rRNA helices (H68–H71) disordered but no HflX, (ii) 50S-HflX complexes with disordered helices H68–H69, and (iii) 50S-HflX complexes with disordered helices H68–H71. Additionally, we observed a distinct HflX–HD conformation in which the apical loop within HD was half bent away from the PTC and landing onto ND1. Notably, the mycobacteria-specific NTE interacted with the ribosomal protein uL16 and 23S rRNA helix 38 at its proximal end, while its distal end extended into the junction region of the disordered rRNA helices H69–71. This finding suggests that the NTE plays a direct role in the disordering of the multiple 23S rRNA helices.

To further substantiate this finding, we prepared a ΔNTE–HflX complex with the ribosome and analyzed it both biochemically and by cryo-EM. While some 70S splitting was observed, the resulting 50S-HflX complex did not exhibit disordered 23S rRNA helices, confirming that the NTE is required for the disordering of H68–H71 during HflX-mediated ribosome splitting (Figure 1B, path 1). Our findings confirmed that, unlike previously proposed mechanisms, in mycobacteria, HflX–ND1 accommodation on the ribosome initiates 70S splitting (Figure 1B, path 2). However, extensive 70S splitting requires HflX–NTE-mediated persistent disordering of multiple 23S rRNA helices, leading to the accumulation of inactive 50S ribosomal subunits (Figure 1B, path 1).

A key question remains: is this the property of HflX, which generates inactive LSUs while dissociating 70S monosomes, unique to mycobacteria or a conserved feature across bacteria? The TRCEM studies of *Eco* and *Lmo* do not report such 50S-HflX classes with disordered 23S rRNA helices [23,24]. It may be argued that in other bacteria, these disordered intermediate 50S states are short-lived and were not captured within the time frame of the TRCEM studies.

HflX is also known for its anti-association activity [12,22]. When we analyzed pre-dissociated *Msm* LSUs bound to *Msm* HflX, the resulting structure closely resembled the 50S-HflX complexes previously reported in *Eco* [12] and *Lmo* [24,29]. In this state, rRNA helices remained intact; H69 was deflected by ~17 Å to avoid a clash with HflX–ND1, the HflX–NTE was unresolved, and the loop within HflX–HD remained extended into the PTC. These findings demonstrate that in *Msm*, HflX employs distinct mechanisms for its ribosomal subunit dissociation and anti-association activities.

Transcriptomics studies indicate that mycobacterial *hflX* expression is regulated by various stress conditions, including antibiotic exposure, nutrient depletion, and chemical stress [30–34]. This raises an important question: what selective advantage does HflX confer by generating inactive LSUs while splitting translationally stalled ribosomes under stress conditions? In both slow- and fast-growing mycobacteria, *hflX* is linked to whiB7, a transcriptional regulator controlling antibiotic resistance and redox homeostasis [21,31,35]. In *Mtb*, *hflX* is overexpressed in response to the hostile macrophage environment [36], while in *Mbo*, *hflX* modulates translational activity, regulating bacterial growth rate and entry into a non-replicating state under hypoxic stress [13]. Bacteria employ diverse mechanisms to remodel the protein synthesis machinery for metabolic down-regulation during latency. Several energy-conservation strategies involve non-canonical translation factors that regulate ribosome activity, such as forming inactive 70S [37] or 100S [38] ribosomes. While 100S ribosome formation has not been reported in mycobacteria, ribosome remodeling in response to stress is well documented [37,39–41]. For instance, under zinc starvation, mycobacterial ribosomes exchange Zn-binding (C+) ribosomal proteins with Zn-free (C−) paralogs [37,39]. Additionally, in the stationary phase, *Msm* accumulates LSUs with an altered H68 conformation, preventing reassociation with SSUs [42].

We speculate that HflX-mediated 70S splitting and disordering of H68–H71 generate a pool of inactive LSUs in response to stress. Since H68–H71 fold in the final stages of LSU biogenesis, it is plausible that upon stress relief, ribosome biogenesis factor(s) rapidly revert inactive LSUs back to functional LSUs to restore translation. Future studies are needed to test this hypothesis.

## Mechanism of HflX-mediated antibiotic resistance

The emergence of antibiotic resistance has rendered many existing antimicrobial agents ineffective, posing a serious challenge in the treatment of tuberculosis and global health in general. One of the mechanisms of antibiotic resistance involves proteins that interact with the drug targets, rescuing them from drug-mediated inhibition of cellular processes [29]. For example, the ribosome protection proteins (RPPs) counteract ribosome-targeting antibiotics through distinct mechanisms—(i) type I RPPs compete with the drug molecule for an overlapping binding site [43–45], (ii) type II RPPs induce allosteric changes in the drug-binding pocket [46–48], and (iii) type III RPPs trigger conformational changes in the target, counteracting the effect of the drug even while it remains bound [49].

While both mycobacteria and *Eco* possess a single copy of the *hflX* gene, *Lmo* carries two distinct copies. The second copy of the *hflX* gene in *Lmo*, *lmo0762*, is induced in response to lincomycin alongside the canonical *hflX* gene (*lmo1296*) [20]. Since *lmo0762* was implicated in lincomycin resistance, it was named *hflXr*, distinguishing it from *lmo1296*, which is implicated to function exclusively in 70S ribosome splitting. Further analysis revealed that some bacteria possess two copies of *hflX*: one with a shorter HD-loop (similar to canonical HflX) and another with a longer HD-loop (similar to HflXr), suggesting distinct functional roles [29]. In *Lmo*, the HflXr variant was proposed to mediate antibiotic resistance through a type II mechanism [29]. In contrast, *Eco*, which has a single copy of *hflX* with a shorter HD-loop, was shown to confer resistance to chloramphenicol [50]. Structural analysis of the *Eco* 50S-HflX complex suggested that the HD-loop of HflX would directly displace chloramphenicol from its binding site, as their binding sites significantly overlap, consistent with a type I mechanism [50].

A previous genetic study demonstrated that HflX-mediated macrolide-lincosamide resistance in *Msm* and *Mab* is comparable to the resistance conferred by erm41, a key macrolide resistance determinant in *Mab* [21]. A recent study in mycobacteria proposed that a conserved nucleotide in the PTC undergoes a conformational change upon erythromycin binding [22]. This altered conformation is recognized by HflX, which subsequently binds tightly to the drug-bound 50S ribosomal subunit, acting as an anti-association factor and preventing its entry into active protein synthesis [22]. However, a key limitation of these studies [22,29,50] is that the proposed mechanisms of antibiotic resistance were inferred from a comparison of two separate structures of either the drug- or the HflX-bound ribosomes, rather than from a single structure of HflX and the antibiotic bound simultaneously to the ribosome.

To address obvious limitations of previous studies [22,29,50], we prepared complexes of 70S ribosomes with HflX together with two PTC-binding antibiotics, erythromycin and chloramphenicol, and determined their structures [25]. These drugs were chosen because a previous study demonstrated that *Mab* and *Msm* Δ*hflX* strains exhibited increased sensitivity to macrolide-lincosamide antibiotics, such as erythromycin, but not to chloramphenicol. From these complexes, we derived four structures: two pre-dissociated 50S-HflX complexes bound to either erythromycin or chloramphenicol, and two 50S-HflX complexes obtained from 70S splitting, each bound to one of the two drug molecules. The distinct structural features of the 23S rRNA helices H68–H71—ordered versus disordered—observed in the 50S subunits derived from complexes prepared between 70S ribosomes and HflX and complexes between pre-dissociated 50S and HflX enabled us to distinguish between drug-bound post-split and pre-dissociated LSUs [25]. As expected, the 50S-HflX drug-bound structures derived from 70S splitting exhibited disordered 23S rRNA helices, H68–H69, while the HD-loop of HflX was retracted away from the PTC, allowing simultaneous HflX and drug binding. Interestingly, in the pre-dissociated 50S-HflX complexes, where the HflX HD-loop remains extended into the PTC, the drugs also remained bound. Moreover, the extended HD-loop, which would overlap with the bound drugs, did not sterically clash with the drug but instead adjusted around it, inducing conformational changes in neighboring PTC nucleotides. These findings clarify that, as proposed for *Lmo* [29] and *Eco* [50], HflX in mycobacteria can bind alongside the drug, thus not mediating antibiotic resistance through either a type I or type II mechanism.

However, this observation raises two important questions: (i) what is the antibiotic resistance mechanism employed by HflX and (ii) why is this resistance specific to the macrolide-lincosamide class of antibiotics? Although our HflX-bound ribosome structures show both macrolide-lincosamide antibiotics and chloramphenicol can bind to the ribosome in the presence of HflX, our structural analysis—supported by a previous biochemical study in *Eco* [12]—reveals that HflX does not bind to ribosomes carrying a peptidyl-tRNA. This is because the presence of a peptidyl-tRNA stabilizes the SSU of the ribosome in an unrotated state, which sterically hinders the accommodation of the HflX–ND1 domain due to a clash with the peptidyl-tRNA. This mechanistic insight helps explain why HflX-mediated resistance in *Msm* is specific to macrolide-lincosamide antibiotics. These drugs promote peptidyl-tRNA drop-off [51–53], generating ribosomal substrates that are compatible with HflX binding and splitting. In contrast, chloramphenicol can bind to ribosomes carrying a peptidyl-tRNA with short nascent peptides (3–6 amino acids) in sequence-specific contexts [54, 55]—conditions that do not favor HflX engagement. As a result, chloramphenicol does not elicit the same HflX-dependent resistance phenotype that is observed with macrolide-lincosamide antibiotics. Furthermore, since we do not observe direct displacement of either of the drugs by HflX in our structures, we propose that the ribosome-splitting activity of HflX is intrinsically linked to its antibiotic resistance phenotype.

**Figure 2:**
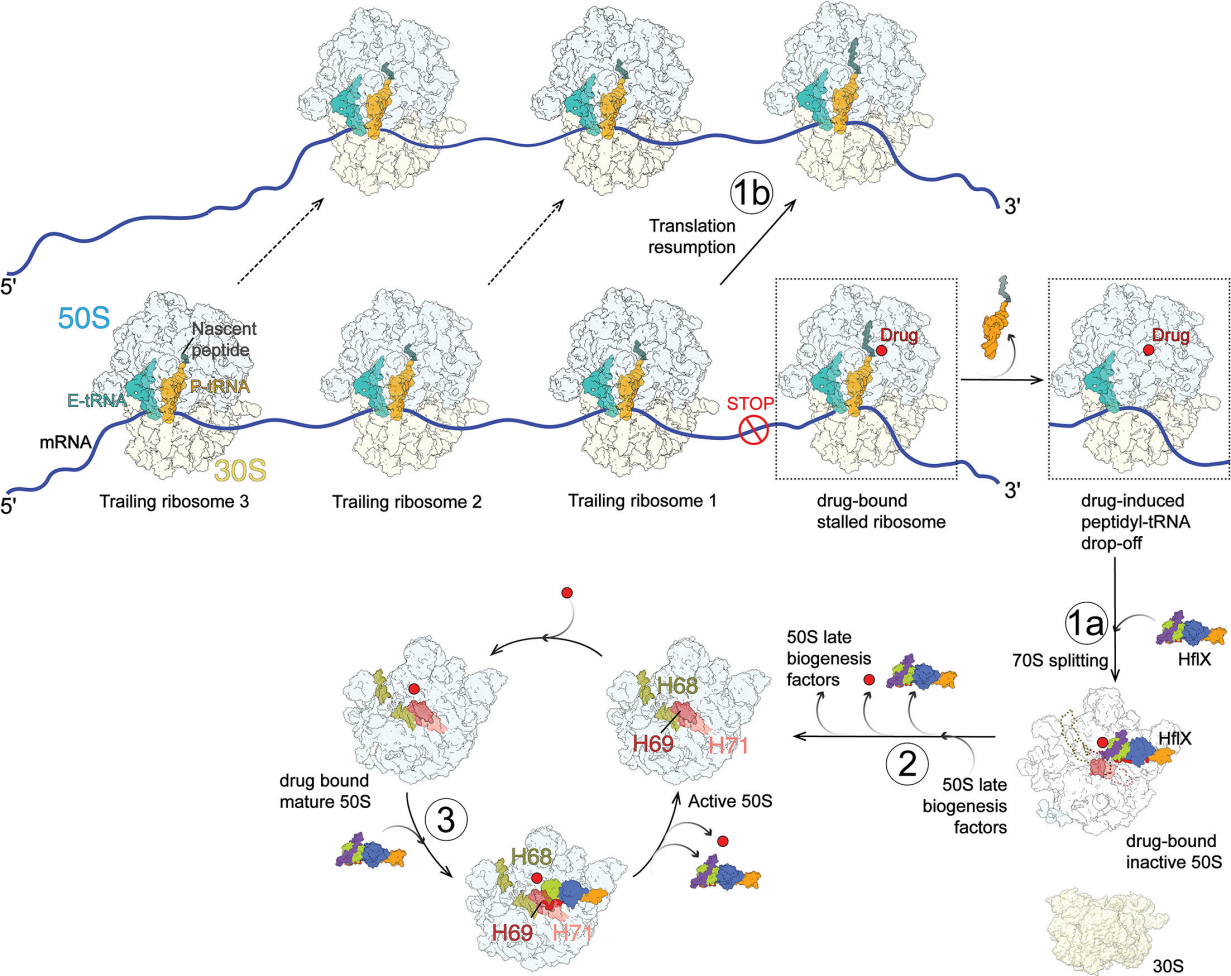
Proposed mechanisms of mycobacterial HflX-mediated macrolide-lincosamide resistance**.** HflX mediates the splitting of drug-stalled 70S ribosomes that have undergone drug-induced peptidyl-tRNA drop-off into 30S and inactive 50S subunits (**1a**), thereby relieves translational roadblocks to allow resumption of translation by trailing ribosomes (**1b**). Drug-bound inactive 50S subunits generated after 70S splitting are subsequently reactivated (**2**) by a ribosome biogenesis factor(s), possibly with concomitant removal of the bound drug. (**3**) HflX binding to drug-bound mature 50S subunit, which may also be referred to as the pre-dissociated 50S, facilitates drug displacement in conjunction with HflX release upon GTP hydrolysis. H68, helix 68; H69, helix 69; H71, helix 71.

Based on our findings, we speculate that HflX may employ the following possible resistance mechanisms (Figure 2): (i) *resolving ribosomal roadblocks in polysomes:* during translation, if a ribosome stalls due to drug binding, trailing ribosomes on the same mRNA also come to a standstill. HflX-mediated splitting of the stalled ribosome could eliminate this roadblock, allowing lagging ribosomes to continue translation; (ii) *reactivating drug-bound inactive LSUs:* splitting of stalled 70S ribosomes generates a pool of inactive, drug-bound LSUs. Subsequent binding of a ribosome biogenesis factor(s) could restore these LSUs to their active state. The bound drug might be removed in the process; and (iii) *facilitating drug removal *via* HflX–HD interactions:* in the pre-dissociated 50S-HflX complexes, we observed interactions between the drug molecule and the HflX HD-loop. It is possible that the HflX dissociation from the LSU also dislodges the drug.

**Perspectives**

- Importance of the field: high frequency of lysogenization X (HflX) is a highly conserved protein that is involved in rescuing drug-stalled ribosomes by imparting macrolide antibiotics resistance in bacteria. An understanding of the molecular mechanism of HflX function is important for developing therapeutics against bacterial infections.
- Current thinking: HflX acts either by directly competing with the drug for an overlapping binding site or by inducing allosteric changes in the drug-binding pocket to release the ribosome-bound drug. Our recent structural study suggests that the mycobacterial HflX imparts drug resistance by removing the drug-bound ribosomal subunits from the active polysome pool, a process that is accompanied by disordering of rRNA of the large ribosomal subunits.
- Future directions: sequence variation among HflXs from different bacterial species, such as N-terminal extensions (NTEs) that are involved in rRNA disordering in mycobacteria, pose three key questions that warrant future investigation: (i) is the observed rRNA disordering activity specific to mycobacterial HflX, or is it a general feature of HflX homologs with NTEs, or does the NTE merely prolong the disordered state to be captured structurally? (ii) since rRNA disordering produces an inactive pool of the drug-bound large ribosomal subunits (LSUs), how are these LSUs brought back to the active pool? (iii) does HflX also play a role in LSU biogenesis in bacteria as reported for its ortholog in mitochondria?

## Abbreviations

CTD: C-terminal domain
*Eco*: *Escherichia coli*
GD: GTPase domain
H69: helix 69
HD: helical domain
HflX: high frequency of lysogenization X
LSU: large ribosomal subunit
*Lmo*: *Listeria monocytogenes*
*Mab*: *Mycobacterium abscessus*
*Mbo*: *Mycobacterium bovis*
*Msm*: *Mycobacterium smegmatis*
*Mtb*: *Mycobacterium tuberculosis*
NTE: N-terminal extension
PTC: peptidyl transferase center
P-site: peptidyl site
RPPs: ribosome protection proteins
Sb: stalk base
TRCEM: time-resolved cryo-EM
